# Correlation of Gut Microbiota Composition with Resistance to Experimental Autoimmune Encephalomyelitis in Rats

**DOI:** 10.3389/fmicb.2016.02005

**Published:** 2016-12-15

**Authors:** Suzana Stanisavljević, Jovanka Lukić, Svetlana Soković, Sanja Mihajlovic, Marija Mostarica Stojković, Djordje Miljković, Natasa Golić

**Affiliations:** ^1^Department of Immunology, Institute for Biological Research “Siniša Stanković," University of BelgradeBelgrade, Serbia; ^2^Laboratory for Molecular Microbiology, Institute of Molecular Genetics and Genetic Engineering, University of BelgradeBelgrade, Serbia; ^3^Institute for Microbiology and Immunology, School of Medicine, University of BelgradeBelgrade, Serbia

**Keywords:** EAE, DGGE, gut microbiota, lactobacilli, *Turicibacter* sp., *Lachnospiraceae*, interleukin-10

## Abstract

Multiple sclerosis is a chronic inflammatory disease of the central nervous system (CNS). It is widely accepted that autoimmune response against the antigens of the CNS is the essential pathogenic force in the disease. It has recently become increasingly appreciated that activated encephalitogenic cells tend to migrate toward gut associated lymphoid tissues (GALTs) and that interrupted balance between regulatory and inflammatory immunity within the GALT might have decisive role in the initiation and propagation of the CNS autoimmunity. Gut microbiota composition and function has the major impact on the balance in the GALT. Thus, our aim was to perform analyses of gut microbiota in experimental autoimmune encephalomyelitis (EAE), an animal model of multiple sclerosis. Albino Oxford (AO) rats that are highly resistant to EAE induction and Dark Agouti (DA) rats that develop EAE after mild immunization were compared for gut microbiota composition in different phases after EAE induction. Microbial analyses of the genus *Lactobacillus* and related lactic acid bacteria showed higher diversity of *Lactobacillus* spp. in EAE-resistant AO rats, while some members of *Firmicutes* and *Proteobacteria* (*Undibacterium oligocarboniphilum*) were detected only in feces of DA rats at the peak of the disease (between 13 and 16 days after induction). Interestingly, in contrast to our previous study where *Turicibacter* sp. was found exclusively in non-immunized AO, but not in DA rats, in this study it was detected in DA rats that remained healthy 16 days after induction, as well as in four of 12 DA rats at the peak of the disease. Similar observation was obtained for the members of *Lachnospiraceae*. Further, production of a typical regulatory cytokine interleukin-10 was compared in GALT cells of AO and DA rats, and higher production was observed in DA rats. Our data contribute to the idea that gut microbiota and GALT considerably influence multiple sclerosis pathogenesis.

## Introduction

Gut microbiota is an essential factor in development of cellular and humoral components of the GALT (Sommer and Bäckhed, 2013), while its dysbiosis have been correlated with various diseases (Carding et al., 2015). Contribution of gut microbiota dysbiosis to the pathogenesis of multiple sclerosis, a chronic inflammatory disease of the CNS is still elusive. There are recent comparative studies on gut microbiota composition in multiple sclerosis patients and healthy subjects that reveal lower abundance of *Faecalibacterium* (Cantarel et al., 2015), *Clostridia* clusters XIVa, IV, *Bacteroides fragilis* and *Sutterella wadsworthensis* (Miyake et al., 2015) *Butyricimonas* (Jangi et al., 2016), and *Parabacteroides*, *Adlercreutzia*, and *Prevotella* genera (Chen et al., 2016) in multiple sclerosis patients. On the contrary, it has been shown that gut content of *Methanobrevibacter* and *Akkermansia* (Jangi et al., 2016), *Pseudomonas*, *Mycoplana*, *Haemophilus*, *Blautia*, and *Dorea* genera (Chen et al., 2016) is increased in multiple sclerosis patients. More data on the effect of gut microbiota on the inflammatory CNS pathology has been obtained from studies on EAE, an animal model of multiple sclerosis. These data support the idea that the gut microbiota dysbiosis is actively contributing to development and progression of multiple sclerosis (Ochoa-Reparaz et al., 2009; Berer et al., 2011; Lee et al., 2011). Moreover, various bacteria and their products have been shown beneficial in EAE, for instance, *B. fragilis* and its capsular polysaccharide A, *Salmonella typhimurium* expressing the CFA/I fimbriae from *E. coli*, *Bifidobacterium animalis*, *Lactobacillus* spp. as well as a probiotic mixture of *Lactobacillus* spp. with *Bifidobacterium bifidum* and *Streptococcus thermophilus* (reviewed in Mielcarz and Kasper, 2015). Gut microbiota-imposed regulation of anti-CNS immune response is performed through generation of tolerogenic dendritic cells and regulatory T cells. Both cell types are induced and propagated in response to various food and microbiota products, including retinoic acid and short chain fatty acids (SCFA), such as butyrate and propionate (Arpaia et al., 2013; Bakdash et al., 2015). Regulatory T cells derived in response to gut bacterial products have been shown efficient in restraining CNS autoimmunity (Ochoa-Repáraz and Kasper, 2016). Hence, the adjustment of the deviated gut microbiota could be a valuable strategy for the prevention and treatment of multiple sclerosis.

Interleukin (IL-10) is a prototypic regulatory cytokine that modulates both innate and adaptive immune response and prevents inappropriate and destructive immune activity as observed in autoimmune disorders (Li and Flavell, 2008). Accordingly, IL-10 knockout mice are more susceptible to induction of EAE (Bettelli et al., 1998), while IL-10 was shown efficient in preventing EAE (Rott et al., 1994; Cua et al., 1999). Importantly, IL-10 is considered as the major immunomodulatory cytokine in the gut microbiota–GALT interaction (Levast et al., 2015). It is produced by both gut parenchyma and GALT cells, including epithelial cells, macrophages, T cells, B cells, dendritic cells, NK cells and innate lymphoid cells (Levast et al., 2015). Notably, its production by GALT regulatory T cells was shown essential for anti-encephalitogenic activity of these cells in EAE (Telesford et al., 2015).

AO rats are highly resistant to EAE induction (Miljkovic et al., 2006), while DA rats develop EAE even after mild immunization (Stosic-Grujicic et al., 2004). We have identified various differences between the strains in peripheral lymphoid organs where anti-CNS immune response is initiated and propagated, as well as in the CNS itself where the immune response is perpetuated and developed into full blown inflammatory response. In general, DA rats, unlike AO rats developed strong autoimmune response, characterized by abundance of IFN-γ-producing T helper (Th)1 cells and IL-17-producing Th17 cells. Consequently, intensive CNS inflammation was observed in DA rats, but not in AO rats (reviewed in Momcilović et al., 2012). In our recent work, AO and DA rats have been compared for their GALT cellular composition and proinflammatory cytokine production as well as for their gut microbiota (Stanisavljević et al., 2016). Differences between the strains within the GALT, including lower percentage of CD4^+^ T cells and reduced generation of IL-17 and IFN-γ in MLN and PP of AO rats were determined (Stanisavljević et al., 2016). Microbial analyses of non-immunized animals have shown higher diversity of *Lactobacillus* spp. in EAE-resistant AO rats comparing to DA rats. Moreover, an uncultivated species of *Turicibacter* genus was found to be exclusively present in feces of AO rats (Stanisavljević et al., 2016).

Here, we present data on the gut microbiota composition in AO and DA rats after EAE induction and we identify potential microbes involved in alleviation of EAE symptoms. Also, production of IL-10, as the major immunoregulatory cytokine, by GALT cells is analyzed in comparison to IL-17 and IFN-γ production.

## Materials and Methods

### Experimental Animals, EAE Induction, and Evaluation

Female AO and DA rats (8–10 weeks of age) were maintained in the animal facility of the Institute for Biological Research “Sinisa Stankovic”. Animal experiments were approved by the local ethics committee (Institute for Biological Research “Sinisa Stankovic”, No. 04-04/15). Housing of the rats was performed under conventional conditions. Three to five rats were kept in the same cage. EAE was induced with rat spinal cord homogenate (SCH) in PBS (50% w/v) mixed with equal volume of CFA (Difco, Detroit, MI, USA). The animals were injected subcutaneously into the hock of one hind limb. The rats were monitored daily for c.s. of EAE, and scored according to the following scale: 0, no c.s.; 1, flaccid tail; 2, hind limb paresis; 3, hind limb paralysis; 4, moribund state or death. DA rats had EAE onset on 9–11 d.p.i. (c.s. 1), peak on 12–16 d.p.i. (c.s. 2–4) and recovery on 18–22 d.p.i. (c.s. 1 or less).

### DGGE Analysis and DNA Sequencing

Data on microbiota composition were derived from three independent experiments. In one of these experiments non-immunized and immunized rats were littermates (AO samples 1–12, DA samples 13–24, as presented in **Figure 2**), while in the remaining experiments AO and DA rats were of the same age and sex (AO samples 25–30, 39, 40, DA samples 31–38, as presented in **Figure 2**). Extraction of bacterial DNA from frozen fecal samples was done using the QIAamp DNA stool minikit (Qiagen, Hilden, Germany). DGGE analysis and gel manipulation after electrophoresis was entirely performed as described previously (Lukic et al., 2013). Lactobacillus-specific primer Lab-0159f paired with the universal reverse primer Uni-0515-GCr (Metabion International, Martinsried, Germany) were used (Heilig et al., 2002). Fragments of interest were excised from the gel and macerated, and the suspension was incubated for 10 min at 98°C (Lukic et al., 2013). After incubation, the suspension was centrifuged to pellet gel particles. The supernatant (30 μl) was used in PCR with Lab-0159f and Uni-0515GCr primers (Heilig et al., 2002). The obtained PCR products were purified using the QIAquick PCR purification kit (Qiagen) and ligated into the pBluescriptT/A vector (Uzelac et al., 2015). Ligated constructs were transformed in Ca_2_-induced competent DH5α cells (Hanahan, 1983), and insert-containing transformants were selected as white colonies on Luria agar (LA) plates containing 100 μg/ml ampicillin and 20 μg/ml X-Gal (5-bromo-4-chloro-3-indolyl-β-D-galactoside) as recommended by Promega. For each excised DNA band, one white colony was picked and plasmids were isolated using the QIAprep spin miniprep kit (Qiagen). The sequencing of the isolated insert-containing pBluescriptT/A plasmids was done with M13F/R primers at Macrogen Europe Service, Amsterdam, Netherlands ^1^. Sequence annotation and the database searches for sequence similarities were performed with the BLAST tool available online^2^.

### Phylogenetic Analysis

The phylogenetic inferences were obtained by MEGA version 7.0 (Kumar et al., 2016). Multiple DNA sequence alignments were performed using Clustal W with default parameters. The construction of a DA/AO gut microbiota phylogenetic tree was conducted by the Maximum Likelihood (ML) method based on the Tamura–Nei model. Bootstrapping of 1000 replicates was used to infer confidence levels of ML tree. The analysis involved 30 nucleotide sequences, 16S rRNA genes obtained by DGGE analysis (see above).

### Isolation of Cells, Cell Culturing, and Generation of Supernatants

Four MLN were isolated from each rat. MLNC were prepared by mechanical disruption of the lymph nodes. PPs were obtained from the small intestine. PPC were obtained by mechanical disruption. The cells were grown in RPMI-1640 medium supplemented with 5% FCS (PAA Laboratories). MLNC (2.5 × 10^6^/ml) and PPC (2 × 10^6^/ml) were stimulated with concanavalin A (ConA, Sigma-Aldrich, 2.5 μg/ml) for 24 h and subsequently cell culture supernatants were collected and kept frozen until assayed.

### ELISA

Cytokine concentration in cell culture supernatants was determined by sandwich ELISA using MaxiSorp plates (Nunc, Rochild, Denmark). For IL-10 detection Rat IL-10 DuoSet ELISA was used according to the manufacturer’s instructions (R&D Systems, Minneapolis, MN, USA). For IFN-γ and IL-17 detection anti-cytokine paired antibodies were used according to the manufacturer’s instructions (eBioscience, San Diego, CA, USA). The antibodies were as follows: anti-rat IFN-γ purified mouse monoclonal (DB1), anti-rat IFN-γ biotinylated rabbit polyclonal, anti-mouse/rat IL-17A purified rat monoclonal (eBio17CK15A5), and anti-mouse/rat IL-17A biotinylated rat monoclonal (eBio17B7). Samples were analyzed in duplicates and the results were calculated using standard curves made on the basis of known concentrations of the recombinant rat IL-10 (R&D Systems) and IFN-γ and IL-17 (Peprotech, Rocky Hill, NJ, USA).

### Statistical Analysis

A Student’s *t*-test (two-tailed) was performed for statistical analysis. A *p*-value less than 0.05 was considered statistically significant.

## Results

### Evaluation of *Lactobacillus* spp. Diversity

**FIGURE 1:**
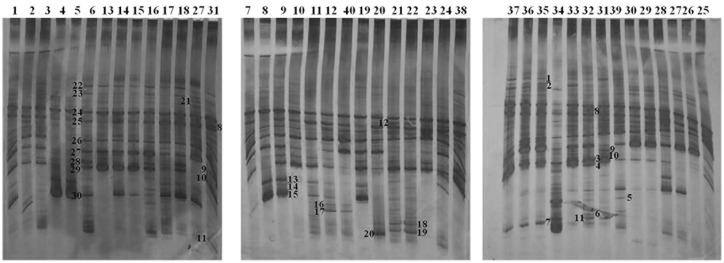
**Denaturing gradient gel electrophoresis profiles of rDNA amplicons obtained using a *Lactobacillus*-specific primer set on bacterial DNA isolated from fecal tissue samples of AO and DA rats.** Each lane represents sample of an individual rat. Total of 40 samples (20 from each of the strains) was analyzed. Distribution of samples between the strains and among different time points is presented in **Figure 2**. Bands indicated by numbers (1–30) were excised, cloned, and sequenced.

**FIGURE 2:**
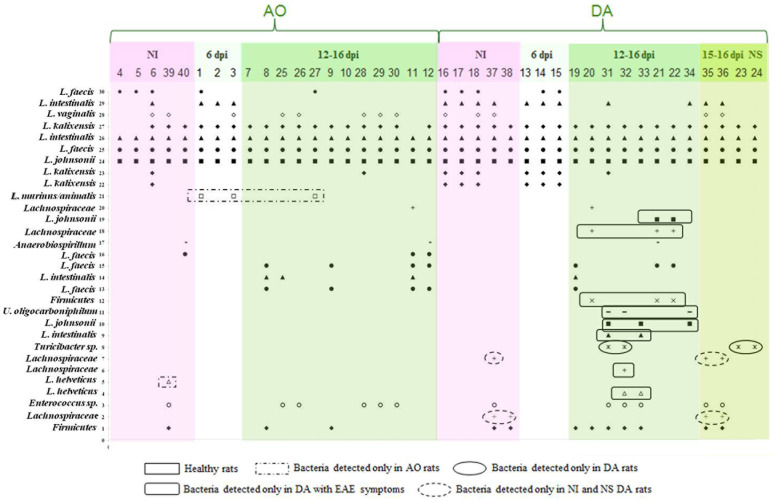
**The identity of the rDNA clones obtained from DGGE bands related to the rats and the period of EAE induction.** The numbers on the *y*-axis (1–30) correlate with the numbers of the bands excised, cloned, and sequenced, while the numbers on the *x*-axis correspond to the numbers of lanes as presented in **Figure 1**. Each lane represents sample of an individual rat. Total of 40 samples (20 from each of the strains) was analyzed. Five samples were collected before the immunization (non-immunized rats – NI), three samples were collected at 6 d.p.i., and 12 samples were collected at 12–16 d.p.i., from each of the strains. There were four samples of DA rats that had no clinical symptoms of EAE (NS).

In order to determine possible microbial players responsible for alleviation of EAE symptoms in DA rats as well as for the EAE-resistance of AO rats, gut microbial diversity was characterized by DGGE analysis of rDNA amplicons using DNA isolated from fecal samples as templates and Lab-0159f and Uni-0515GCr primer set. In total, 30 unique DNA fragment bands (16 from AO and 14 from DA rat fecal samples) have been cloned and sequenced (**Figure 1**). The sequence analysis revealed that most of the bands (19/30) belonged to *Lactobacillus* species (99–100% nucleotide sequences identity) (**Table 1**). Among 19 sequences belonging to *Lactobacillus* sp. the most abundant were *Lactobacillus kalixensis*, *L. johnsonii*, *L. intestinalis*, and *L. faecis* that were detected in all samples, presumably constituting the core measurable microbiota (**Figure 2**; **Table 1**). *L. helveticus*, *L. murinus/animalis*, and *L. vaginalis* as well as *Enterococcus* sp. were sporadically present in AO and DA rats, both healthy and with EAE symptoms.

**Table 1:** Clones with the percentage of identity to known sequences in BLAST database.

No. of band	Species^a^	NSI (%)
1	Uncultured Firmicutes bacterium clone TM1-142 16S ribosomal RNA gene, partial sequence	98
2	Uncultured *Lachnospiraceae* bacterium clone MS051A1_A08 16S ribosomal RNA gene	95
	Uncultured Clostridiales bacterium gene for 16S rRNA, partial sequence, clone: M_Fe_Clo047	95
3	*Enterococcus faecium/faecalis/durans*	93
	*Lactobacillus casei* strain A5	
4	*Lactobacillus helveticus*	99
5	*Lactobacillus helveticus*	99
6	Uncultured *Lachnospiraceae* bacterium clone 78 16S ribosomal RNA gene	99
	Uncultured Firmicutes bacterium clone CM2-40 16S ribosomal RNA gene, partial sequence	99
7	Uncultured *Lachnospiraceae* bacterium clone MS051A1_A08 16S ribosomal RNA gene	96
	Uncultured Clostridiales bacterium gene for 16S rRNA, partial sequence, clone: M_Fe_Clo047	95
8	*Turicibacter* sp. LA62	98
9	*Lactobacillus intestinalis* TH4	100
10	*Lactobacillus johnsonii* 17c	99
11	*Burkholderiales* bacterium clone Cat004D_G05 (Proteobacteria)	99
	*Undibacterium oligocarboniphilum* strain EM 1	
12	Uncultured Firmicutes bacterium clone CTF1-97	100
13	*Lactobacillus faecis* FZB1	100
14	*Lactobacillus intestinalis* TH4	99
15	*Lactobacillus faecis* FZB1	100
16	*Lactobacillus faecis* FZB1	99
17	Uncultured Proteobacteria clone TCM2-12	99
	*Anaerobiospirillum* sp. (Proteobacteria)	95
18	Uncultured Firmicutes bacterium clone TCF2-116	92
	Uncultured *Lachnospiraceae* bacterium clone FecI012	92 (3% gaps)
19	*Lactobacillus johnsonii* 17c	99
20	Uncultured *Lachnospiraceae* bacterium clone MS051A1_A08	96
	Uncultured Clostridiales bacterium gene for 16S rRNA	95
21	*Lactobacillus murinus*	100
	*Lactobacillus animalis* TSU4	100
22	*Lactobacillus kalixensis* CCUG 48459	100
	*Lactobacillus intestinalis*	95
23	*Lactobacillus kalixensis* CCUG 48459	100
24	*Lactobacillus johnsonii* 17c	99
25	*Lactobacillus faecis* FZB1	99
26	*Lactobacillus intestinalis* TH4	100
27	*Lactobacillus kalixensis* CCUG 48459	100
28	*Lactobacillus vaginalis*	99
29	*Lactobacillus intestinalis* TH4	99
30	*Lactobacillus faecis* FZB1	99

*^a^Species were named according to their closest relative. NSI, nucleotide sequence identity.*

Further, the presence of specific bands in DGGE profiles of AO and DA rats was evaluated using Fisher’s exact test, where only the clearly visible bands were counted. Results of Fisher’s exact test revealed the presence of *Lachnospiraceae* exclusively in DA rats, regardless of immunization, both in healthy rats and those with EAE symptoms. Similarly, bacteria belonging to *Turicibacter* sp. were detected in DA rats both healthy and with EAE symptoms, but not in non-immunized DA rats. On the other hand, one group of bacteria belonging to *Firmicutes* and bacteria belonging to *Proteobacteria* (*Burkholderiales*, *Undibacterium oligocarboniphilum*) were detected only in DA rats from 12 to 15 d.p.i. in the peak of the disease (**Figure 2**; **Table 2**). In general, in EAE-resistant healthy AO rats only lactobacilli and enterococci were detected, except in the case of two AO rats (non-immunized and 16 d.p.i.) where *Anaerobiospirillum* was detected together with an immunized DA rat (15 d.p.i.).

**Table 2:** The abundance of bands indicated by numbers (1–30) in **Figure 1** (given in percentage) in AO and DA rats and in different clinical status.

		AO			DA		
Band no.	%	NI (5)	6 dpi (3)	12–16 dpi (12)	NI (5)	6 dpi (3)	12–16 dpi (12)
1	*Firmicutes*	20	0	16,67	40	0	58,33
2	*Lachnospiraceae* (2)	0	0	0	40	0	16,67
3	*Enterococcus* sp. (3)	20	0	41,67	20	0	41,67
4	*L. helveticus* (4)	0	0	0	0	0	16,67
5	*L. helveticus* (5)	20	0	0	0	0	0
6	*Lachnospiraceae* (6)	0	0	0	0	0	8,33
7	*Lachnospiraceae* (7)	0	0	0	20	0	16,67
8	*Turicibacter* (8)	0	0	0	0	0	33,33
9	*L. intestinalis* (*9*)	0	0	0	0	0	16,67
10	*L. johnsonii* (*10*)	0	0	0	0	0	25
11	*Undibacterium oligocarboniphilum* (11)	0	0	0	0	0	25
12	*Firmicutes* (12)	0	0	0	0	0	25
13	*L. faecis* (*13*)	0	0	33,33	0	0	8,33
14	*L. intestinalis* (14)	0	0	25	0	0	8,33
15	*L. faecis* (15)	0	0	33,33	0	0	25
16	*L. faecis* (16)	20	0	16,67	0	0	0
17	*Anaerobiospirillum* (17)	20	0	8,33	0	0	8,33
18	*Lachnospiraceae* (18)	0	0	0	0	0	25
19	*L. johnsonii* (19)	0	0	0	0	0	16,67
20	*Lachnospiraceae* (20)	0	0	8,33	0	0	8,33
21	*L. murinus/animalis* (21)	0	66,67	8,33	0	0	0
22	*L. kalixensis* (*22*)	20	0	0	60	100	0
23	*L. kalixensis* (23)	20	0	8,33	60	100	8,33
24	*L. johnsonii* (24)	100	100	100	100	100	100
25	*L. faecis* (25)	100	100	100	100	100	100
26	*L. intestinalis* (26)	100	100	100	100	100	100
27	*L. kalixensis* (27)	60	100	91,67	100	100	100
28	*L. vaginalis* (28)	40	33,33	41,67	60	0	8,67
29	*L. intestinalis* (29)	20	100	0	80	100	33,33
30	*L. faecis* (30)	60	33,33	8,33	60	66,67	0

*The band numbers correspond to band numbers given in **Figure 1**.*

**FIGURE 3:**
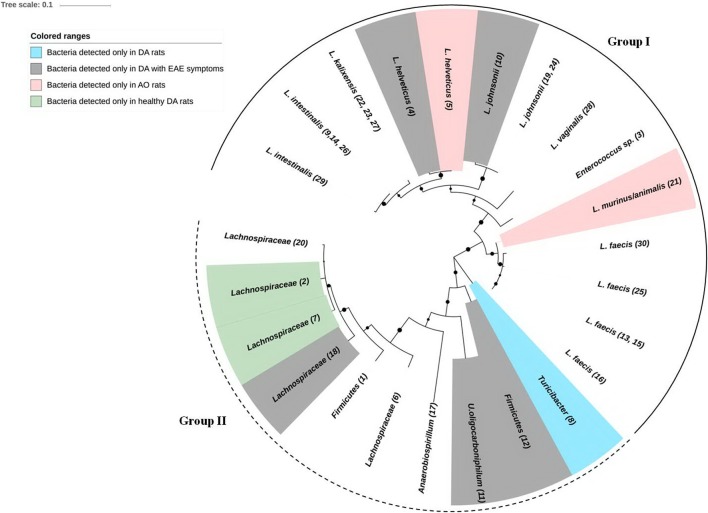
**Phylogenetic inferences of 16S rRNA gene among bacteria isolated from DA and AO feces samples.** A phylogenetic tree of 16S rRNA genes was constructed with the maximum likelihood (ML) method using a Tamura–Nei model distance matrix. The confidence levels were calculated from 1000 bootstrap resamples of alignment used for phylogenetic inferences by ML method. Black circles represent the nodes with a support bootstrap value of ≥40%. Numbers in brackets represent the number of corresponding excised, cloned, and sequenced DGGE band. Group I, including phylum *Firmicutes* and bacteria from the families *Lactobacillaceae*, *Enterococcaceae*, and *Turicibacteraceae* is denoted by full semicircular line. Group II consisting of bacteria belonging to phylum *Firmicutes* and *Proteobacteria* including families *Lachnospiraceae*, *Oxalobacteraceae*, and *Succinivibrionaceae* is denoted by dashed semicircular line.

Finally, ML phylogenetic analysis separated DA/AO gut microbiota into two distinct groups (**Figure 3**). Larger group, the Group I includes phylum *Firmicutes* and bacteria from the families *Lactobacillaceae*, *Enterococcaceae*, and *Turicibacteraceae*. Smaller and less conserved group, the Group II consists of bacteria belonging to phylum *Firmicutes* and Proteobacteria including families *Lachnospiraceae*, *Oxalobacteraceae*, and *Succinivibrionaceae*. Interestingly, the results of ML phylogenetic analysis revealed that the clones 2 and 7 (**Figure 1**; **Table 1**) detected only in healthy DA rats belong to the same phylogenetic group within family *Lachnospiraceae*, possibly the same species (**Figure 3**). In contrast the clones 6 and 18 that were only detected in DA rats with EAE symptoms according to ML phylogeny analysis possibly belonging to different species of the family *Lachnospiraceae*.

### IL-10 Production in MLNC of AO and DA Rats

**FIGURE 4:**
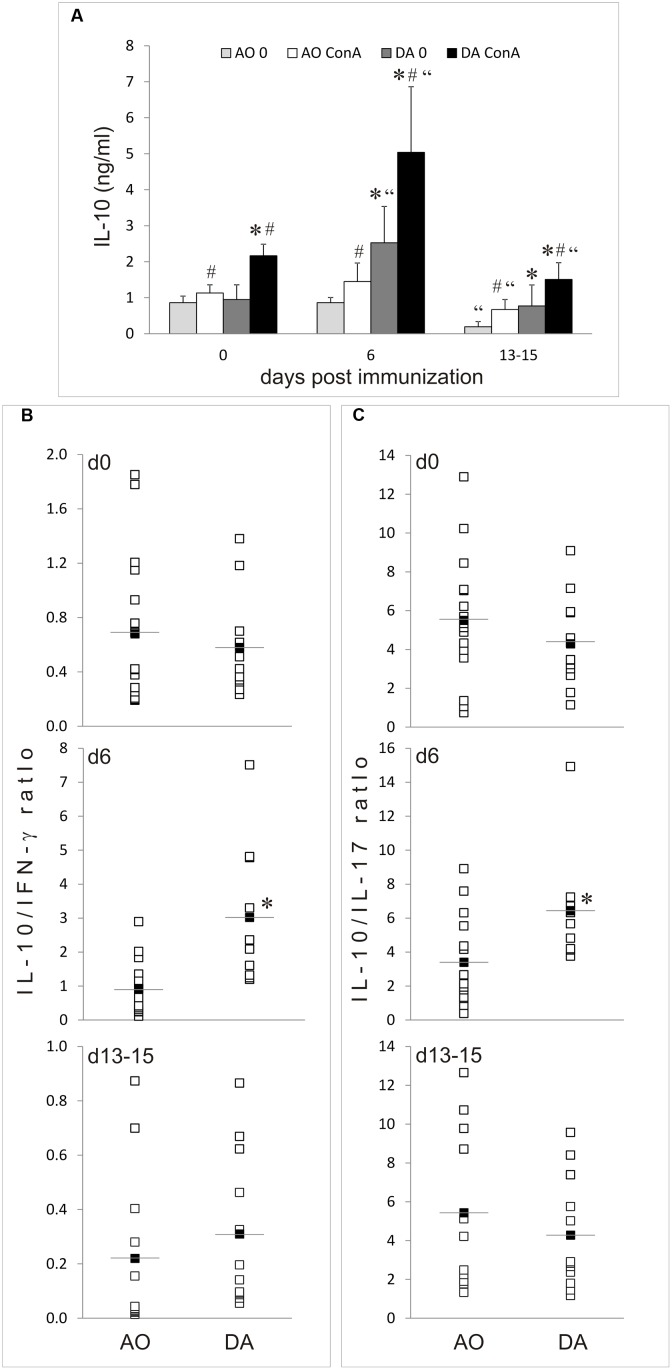
**IL-10 production in MLNC.** MLNC were isolated from non-immunized (0 d.p.i.) AO and DA rats or from immunized rats at 6 d.p.i and 13–15 d.p.i. Cytokine levels were measured in supernatants of 24 h cultures of un-stimulated (0) or ConA-stimulated (ConA) cells. IL-10 levels are presented as mean ± SD from at least six rats per group **(A)**. Ratios of IL-10 levels to IFN-γ **(B)** or to IL-17 **(C)** levels determined in ConA-stimulated cultures are presented as individual values (white squares) and as mean (black squares with line). ^∗^*p* < 0.05 AO vs. DA; “*p* < 0.05 to 0 d.p.i. of the same strain; ^#^*p* < 0.05 0 vs. ConA.

IL-10 production was determined in cultures of MLNC obtained from non-immunized AO and DA rats (day 0) and at day 6 and days 13–16 after the immunization. IL-10 release was similar in untreated AO rats on day 0 and day 6, but then decreased on days 13–16 (**Figure 4A**). On the contrary, spontaneous IL-10 generation increased on day 6 in DA rats and then returned to basal levels on days 13–15 (**Figure 4A**). ConA stimulated IL-10 generation in MLNC of both strains in all groups of samples and consequently similar strain-specific pattern of IL-10 production was observed in ConA-stimulated MLNC as in unstimulated cultures. Interestingly, IL-10 production was significantly higher in DA rat than in AO rat samples in all of the analyzed samples, except for non-stimulated cultures of day 0. IFN-γ and IL-17 production were analyzed in parallel. The same pattern of IFN-γ and IL-17 production was observed as in our previous study (Stanisavljević et al., 2016). In order to get insight into IL-10 production relative to production of these major pro-inflammatory cytokines, ratios of IL-10 to IFN-γ and IL-17 were calculated for each sample. The only significant difference between the strains was observed with day 6 samples, where both IL-10/IFN-γ and IL-10/IL-17 ratios were higher in DA rats than in AO rats (**Figures 4B,C**). Thus, it is clear that EAE-resistant and EAE-prone rats have different regulation of IL-10 production in MLN both in non-immunized and EAE rats.

### IL-10 Production in PPC of AO and DA Rats

**FIGURE 5:**
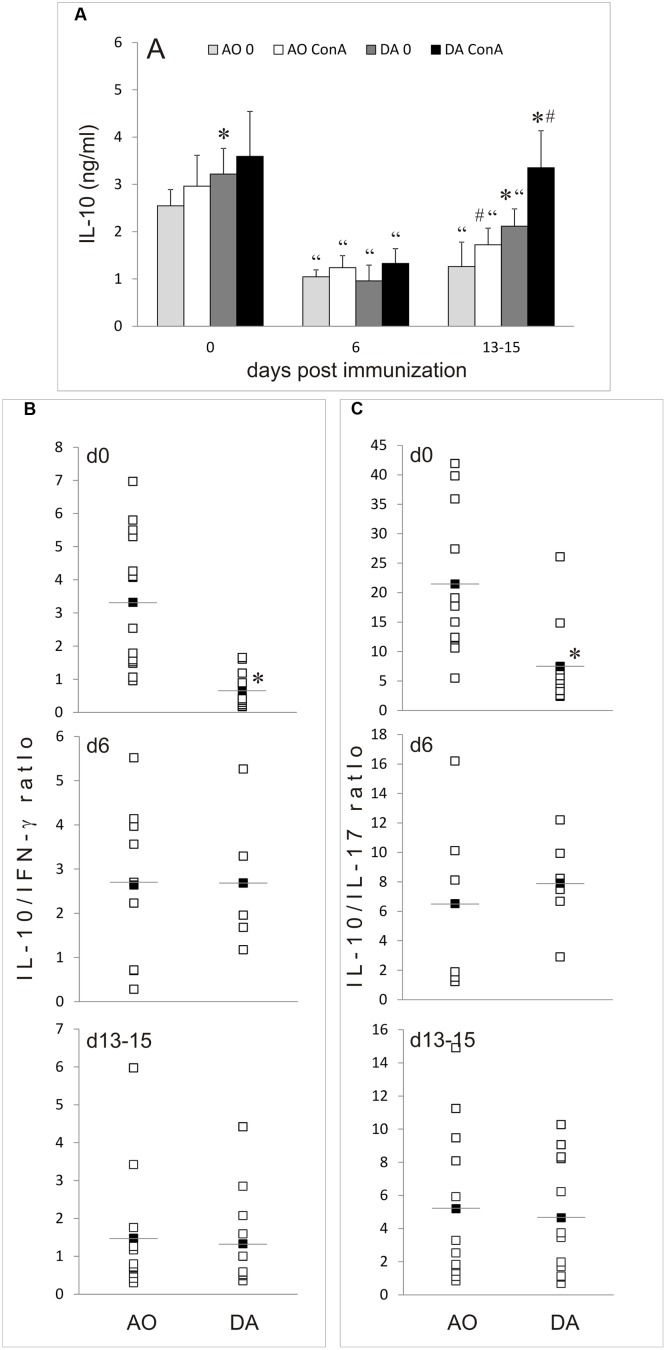
**IL-10 production in PPC.** PPC were isolated from non-immunized (0 d.p.i.) AO and DA rats or from immunized rats at 6 d.p.i and 13–15 d.p.i. Cytokine levels were measured in supernatants of 24 h cultures of un-stimulated (0) or ConA-stimulated (ConA) cells. IL-10 levels are presented as mean ± SD from at least six rats per group **(A)**. Ratios of IL-10 levels to IFN-γ **(B)** or to IL-17 **(C)** levels determined in ConA-stimulated cultures are presented as individual values (white squares) and as mean (black squares with line). ^∗^*p* < 0.05 AO vs. DA; “*p* < 0.05 to 0 d.p.i. of the same strain; ^#^*p* < 0.05 0 vs. ConA.

IL-10 production was determined in cultures of PPC obtained from non-immunized AO and DA rats (day 0) and at day 6 and days 13–16 after the immunization. Spontaneous and ConA-stimulated IL-10 release was higher in non-immunized rats of both strains than on day 6 or days 13–16 (**Figure 5A**). The only exemption was ConA stimulated IL-10 generation in PPC of DA rats on days 13–16 which was similar to the level observed in samples of non-immunized counterparts. Generally, ConA was inefficient in inducing IL-10 production in PPC of both strains, with an exemption with DA samples obtained on days 13–16. Strain differences were observed only on days 13–15 for both spontaneous and ConA-induced production of the cytokine (**Figure 5A**). As for the ratios of IL-10 to IFN-γ and IL-17, the only significant difference between the strains was observed with samples obtained from non-immunized rats, where both IL-10/IFN-γ and IL-10/IL-17 ratios were higher in AO rats (**Figures 5B,C**). Thus, these results imply that EAE-resistant and EAE-prone rats have different regulation of IL-10 production in PPC.

## Discussion

Gut microbiota composition is different in multiple sclerosis patients and healthy controls (Cantarel et al., 2015; Miyake et al., 2015; Chen et al., 2016; Jangi et al., 2016). Accordingly, EAE-prone and EAE-resistant rats are shown to differ for gut microbiota composition in our study. Specifically, *Turicibacter* sp. and the members of *Lachnospiraceae* family are identified as possible EAE resistance/recovery promoters. Also, production of IL-10 as the major gut immunoregulatory cytokine is diverse between the rat strains. Our results imply that microbiota–GALT interaction differs in the rat strains and that this dissimilarity could be important for their susceptibility/resistance to the CNS autoimmunity.

It could be referred that gut microbiota composition of AO rats is more stable, representing core measurable microbiota, while the results obtained for DA rats point to higher diversity of bacterial groups, especially at the peak of the diseases. However, the higher diversity of bacteria in DA rats could be result of lower number of lactobacilli as dominant groups in DA rats. Thus, other microbial groups overgrow due to the lack of lactobacilli. According to Benson et al. (2010), *Turicibacter* sp. constitutes the core measurable microbiota in mice and it was suggested that its quantitative variations were related to the host genotype. The increased *Turicibacter* gut content was correlated with increase in butyric acid, a short chain fatty acid (SCFA) with immunomodulatory potential (Zhong et al., 2015). In addition, possible anti-inflammatory effects of *Turicibacter* were suggested (Presley et al., 2010; Werner et al., 2011; Suchodolski et al., 2012). In our previous study, when non-immunized AO and DA rats were compared, *Turicibacter* was detected only in feces of AO rats (Stanisavljević et al., 2016). In contrast to previous results, in this study *Turicibacter* sp. was not detected in AO, but only in DA rats, in samples obtained at the time of EAE peak when higher production of IL-10 by MLNC and PPC was also observed. The discrepancy of the obtained results could be explained by the inter-individual microbial diversity (Guinane and Cotter, 2013), as well as by limitations of molecular method used in the study which allows detection of small percent (1%) of dominant microbial groups present in samples at the time of sampling. It has been established that only part of the microbial community members are stabile over time and participate in individual core microbial population, pointing to the conclusion that *Turicibacter* possibly does not belong to the core microbiota in the rats used in our studies. On the other hands, a number of environmental and host factors that are still not well known could cause the observed changes. Nevertheless, the obtained results could be a good indication for future studies. As our data do not allow adequate statistical analysis on the correlation between IL-10 generation and the presence of *Turicibacter* sp. in the gut microbiota, it is on the future studies to determine if there is mutual stimulatory effect between IL-10 and *Turicibacter* sp. Also, future studies should determine if some other host factors stimulate the presence of Turicibacter sp. in DA rats at the peak of the disease.

In addition, the results of this study revealed that members of the family *Lachnospiraceae* could be linked to the EAE alleviation. It was reported previously that bacteria belonging to the family *Lachnospiraceae* were dominantly present in the gut microbiota in *Clostridium difficile*-infected animals with mild disease (Reeves et al., 2011). The authors hypothesized that members of the *Lachnospiraceae* family enabled partially restored colonization resistance against *Clostridium difficile* in the murine gut. Further, decreased prevalence of *Lachnospiraceae* in gut microbiota of patients with inflammatory bowel disease (IBD) was reported (Frank et al., 2007). *Lachnospiraceae* are Gram-positive obligate anaerobes that are mostly non-spore forming (Cotta and Forster, 2006). Interestingly, the clones 2, 7, and 20, identified as members of *Lachnospiraceae* family, showed higher similarity to *Clostridiales*. Taxon *Clostridiales* is a bacterial order of the phylum *Firmicutes* with important roles in the colonic fermentation of dietary fiber (Chinda et al., 2004). Moreover, bacteria belonging to order *Clostridiales*, especially members of family *Lachnospiraceae*, were identified as the most active microbial components in the gut of healthy adults and strongly contribute to production of beneficial SCFAs in the gut (Chinda et al., 2004; Peris-Bondia et al., 2011; Leonel and Alvarez-Leite, 2012). Hence, it could be hypothesized that the increase in *Clostridiales* and *Lachnospiraceae* in healthy DA rats is linked to increased production of intestinal butyrate associated with better healthy status.

Generally, large number of factors influence the establishment and maintenance of microbiota composition, including host genetic background, environment, immune response, as well as microbe–microbe interactions (Spor et al., 2011). AO and DA rats have different major histocompatibility complex (MHC) haplotype, RT1u and RT1av1, respectively. Since rats of both strains are kept under identical environmental conditions in our studies, it is likely that MHC disparity contributes to established difference in AO and DA gut microbiota. Further, it has recently been shown that fecal micro RNA (miRNA) facilitates host control of the gut microbiota through miRNA-mediated inter-species gene regulation (Liu et al., 2015). Hence the possible involvement of miRNA in control of the microbiota composition in AO and DA rats upon EAE induction will be the subject of our further studies.

The observed variation in gut microbiota composition could largely contribute to differential regulatory GALT status in AO and DA rats. Different microbes and their products could potentiate or restrict generation of tolerogenic dendritic cells and regulatory T cells in the GALT. These cells can regulate encephalitogenic immune response locally, as it has been shown that encephalitogenic T cells tend to migrate into the GALT (Berer et al., 2014). Also, regulatory T cells can migrate into the CNS where they restrict encephalitogenic T cell proliferation, cytokine production and other effector functions (Koutrolos et al., 2014). Importantly, IL-10-producing regulatory T cells have been identified as the primary anti-encephalitogenic population in mice treated with *B. fragilis* capsular polysaccharide A (Telesford et al., 2015). IL-10 is one of the major regulatory cytokines of the immune system and it has a profound role in microbiota-imposed immunoregulation within the GALT (Levast et al., 2015). Although macrophages and B cells are considered as the most potent producers of IL-10 within GALT, it seems that IL-10 produced by regulatory T cells has a dominant role in building a regulatory milieu in the gut tissue (reviewed in Guo, 2016). Indeed, it was shown that the native human gut microorganisms, mainly clusters IV and XIVa of the genus *Clostridium* stimulate accumulation of IL-10-producing regulatory T cells in the gut (Atarashi et al., 2011). Importantly, *Clostridia* clusters IV and XIVa were found depleted in multiple sclerosis patients (Miyake et al., 2015). Noteworthy, our previous study showed that proportion of FoxP3^+^ regulatory T cells increased within MLNC and PPC of rats at 13–15 d.p.i. (Stanisavljević et al., 2016). This increase might, at least partly, contribute to augmented production of IL-10 in DA rat GALT at the peak of EAE.

Higher production of IL-10 was observed in draining lymph nodes of DA rats in comparison to AO rats at day 6 after the immunization in our previous study (Blaževski et al., 2013). Similar results were obtained with MLN in this study: IL-10 release was higher in DA rats before the immunization, as well as at day 6 and days 13–16 after the immunization. Actually, generation of IL-10 in AO rats remained at the basal levels after the immunization, while it increased in DA rats at day 6 and then declined on days 13–16. Interestingly, IL-10 production decreased after the immunization in DA rat PPC and then increased toward basal levels on days 13–16. This implies that redistribution of IL-10-generating cells between PP and MLN or even draining lymph nodes might occur in DA rats after EAE induction. Indeed, our previous results showed that there were changes in cellular composition of MLN and PP from non-immunized toward EAE rats (Stanisavljević et al., 2016). Specifically, proportion of CD4^+^ T cells among MLN and PP decreased upon immunization in both strains. Also, percentage of CD4^+^ T cells among MLN and PP was higher in DA than in AO rats. These changes might contribute to differential production of IFN-γ, IL-17, and IL-10 observed in our studies. Also, as absolute number of cells per MLN and PP did not differ between non-immunized and immunized rats (Stanisavljević et al., 2016) the changes in cellular composition imply selective migration of certain cell types to/from MLN and PP. Alternatively, some IL-10-promoting factors could work within MLN and some IL-10 inhibitory elements within PP of immunized DA rats. These two mechanisms are not mutually exclusive and could act in cooperation to modulate IL-10 release in GALT. Further, ratio of IL-10 to proinflammatory cytokine production is higher in AO rats than in DA rats in PPC before the immunization. This implies that basal milieu in PPC of EAE-resistant rats is more immunoregulatory than in EAE-prone rats. The same trend is observed in MLN, yet without statistical difference. However, the ratio is higher in DA rats MLN at day 6 after the immunization thus implying that intensive immunoregulatory activity is present within MLN of EAE-prone rats in the inductive phase of the disease. Detailed studies on the functional significance and mechanisms of the observed modulation of IL-10 production during the course of EAE are warranted.

## Conclusion

In this study we have analyzed the gut microbiota composition in AO and DA rats after EAE induction. As a result, *Turicibacter* sp. and the members of *Lachnospiraceae* family were identified as possible EAE-modulating bacteria, while strain specificities between AO and DA rats in GALT IL-10 generation were observed. Our results contribute to a view that functional studies aiming at altering gut microbiota and gut-associated immune response as the primary tool to reduce encephalitogenic immune response in multiple sclerosis are needed.

## Ethics Statement

This study was approved by Ethics committee of the Institute for Biological Research “Sinisa Stankovic.” Compliance with Directive 2010/63/EU on the protection of animals used for experimental and other scientific purposes.

## Author Contributions

SuS: Performed main work, analyzed, interpreted, and critically revised the data; JL: DGGE analysis, performed work and made part of the draft related to DGGE analysis; SvS: Performed the DGGE analysis, sequencing, and analysis of the sequenced data; SM: Conception and design of phylogenetic analysis, analyzed, interpreted, and critically revised the data; MS: Conception and design related to immunology and critically revised the manuscript; DM: Immunology: conception and design, supervised the work, analyzed and interpreted the data, draft the work, and critically revised the manuscript; NG: Microbiology: conception and design, supervised the work, analyzed and interpreted the data, draft the work, and critically revised the manuscript; all authors finally approved the version to be published and agreed to be accountable for all aspects of the work in ensuring that questions related to the accuracy or integrity of any part of the work are appropriately investigated and resolved.

## Conflict of Interest Statement

The authors declare that the research was conducted in the absence of any commercial or financial relationships that could be construed as a potential conflict of interest.

## Footnotes

1 https://dna.macrogen.com/eng/support/ces/guide/universal_primer.jsp

2 http://blast.ncbi.nlm.nih.gov/Blast.cgi

## Abbreviations

AO: Albino Oxford
c.s.: clinical score
CFA: complete Freund’s adjuvant
CNS: central nervous system
d.p.i.: days post immunization
DA: Dark Agouti
DGGE: denaturing gradient gel electrophoresis
EAE: experimental autoimmune encephalomyelitis
FCS: fetal calf serum
GALT: gut associated lymphoid tissue
IFN: interferon
IL: interleukin
MBP: myelin basic protein
MLN: mesenteric lymph node
MLNC: MLN cells
NSI: nucleotide sequence identity
PBS: phosphate buffer saline
PCR: polymerase chain reaction
PP: Peyer’s patch
PPC: PP cells
SD: standard deviation
Th: helper T cells.

## References

[B1] Arpaia N. Campbell C. Fan X. Dikiy S. van der Veeken J. deRoos P. (2013). Metabolites produced by commensal bacteria promote peripheral regulatory T-cell generation. *Nature* 504 451–455. 10.1038/nature12726 24226773 PMC3869884

[B2] Atarashi K. Tanoue T. Shima T. Imaoka A. Kuwahara T. Momose Y. (2011). Induction of colonic regulatory T cells by indigenous *Clostridium* species. *Science* 331 337–341. 10.1126/science.1198469 21205640 PMC3969237

[B3] Bakdash G. Vogelpoel L. T. van Capel T. M. Kapsenberg M. L. de Jong E. C. (2015). Retinoic acid primes human dendritic cells to induce gut-homing, IL-10-producing regulatory T cells. *Mucosal Immunol.* 8 265–278. 10.1038/mi.2014.64 25027601

[B4] Benson A. K. Kelly S. A. Legge R. Ma F. Low S. J. Kim J. (2010). Individuality in gut microbiota composition is a complex polygenic trait shaped by multiple environmental and host genetic factors. *Proc. Natl. Acad. Sci. U.S.A.* 107 18933–18938. 10.1073/pnas.1007028107 20937875 PMC2973891

[B5] Berer K. Boziki M. Krishnamoorthy G. (2014). Selective accumulation of pro-inflammatory T cells in the intestine contributes to the resistance to autoimmune demyelinating disease. *PLoS ONE* 9:e87876 10.1371/journal.pone.0087876 PMC3913661 24504092

[B6] Berer K. Mues M. Koutrolos M. Rasbi Z. A. Boziki M. Johner C. (2011). Commensal microbiota and myelin autoantigen cooperate to trigger autoimmune demyelination. *Nature* 479 538–541. 10.1038/nature10554 22031325

[B7] Bettelli E. Das M. P. Howard E. D. Weiner H. L. Sobel R. A. Kuchroo V. K. (1998). IL-10 is critical in the regulation of autoimmune encephalomyelitis as demonstrated by studies of IL-10- and IL-4-deficient and transgenic mice. *J. Immunol.* 161 3299–3306. 9759845

[B8] Blaževski J. Petković F. Momèilović M. Jevtic B. Miljković D. Mostarica Stojković M. (2013). High interleukin-10 expression within the central nervous system may be important for initiation of recovery of Dark Agouti rats from experimental autoimmune encephalomyelitis. *Immunobiology.* 218 1192–1199. 10.1016/j.imbio.2013.04.004 23664544

[B9] Cantarel B. L. Waubant E. Chehoud C. Kuczynski J. DeSantis T. Z. Warrington J. (2015). Gut microbiota in multiple sclerosis: possible influence of immunomodulators. *J. Investig. Med.* 63 729–734. 10.1097/JIM.0000000000000192 PMC4439263 25775034

[B10] Carding S. Verbeke K. Vipond D. T. Corfe B. M. Owen L. J. (2015). Dysbiosis of the gut microbiota in disease. *Microb. Ecol. Health Dis.* 26:26191 10.3402/mehd.v26.26191 PMC4315779 25651997

[B11] Chen J. Chia N. Kalari K. R. Yao J. Z. Novotna M. Soldan M. M. (2016). Multiple sclerosis patients have a distinct gut microbiota compared to healthy controls. *Sci Rep.* 6:28484 10.1038/srep28484 PMC4921909 27346372

[B12] Chinda D. Nakaji S. Fukuda S. Sakamoto J. Shimoyama T. Nakamura T. (2004). The fermentation of different dietary fibers is associated with fecal clostridia levels in men. *J. Nutr.* 134 1881–1886. 15284370 10.1093/jn/134.8.1881

[B13] Cotta M. Forster R. (2006). The family Lachnospiraceae, including thegenera *Butyrivibrio*, *Lachnospira* and Rosburia. *Prokaryotes* 4 1002–1021.

[B14] Cua D. J. Groux H. Hinton D. R. Stohlman S. A. Coffman R. L. (1999). Transgenic interleukin 10 prevents induction of experimental autoimmune encephalomyelitis. *J. Exp. Med.* 189 1005–1010. 10.1084/jem.189.6.1005 10075984 PMC2193046

[B15] Frank D. N. St. Amand A. L. Feldman R. A. Boedeker E. C. Harpaz N. Pace N. R. (2007). Molecular-phylogenetic characterization of microbial community imbalances in human inflammatory bowel diseases. *Proc. Natl. Acad. Sci. U.S.A.* 104 13780–13785. 10.1073/pnas.0706625104 17699621 PMC1959459

[B16] Guinane C. M. Cotter P. D. (2013). Role of the gut microbiota in health and chronic gastrointestinal disease: understanding a hidden metabolic organ. *Ther. Adv. Gastroenterol.* 6 295–308. 10.1177/1756283X13482996 PMC3667473 23814609

[B17] Guo B. (2016). IL-10 modulates Th17 pathogenicity during autoimmune diseases. *J. Clin. Cell Immunol.* 7:400 10.4172/2155-9899.1000400 PMC4905582 27308096

[B18] Hanahan D. (1983). Studies on transformation of *Escherichia coli* with plasmids. *J. Mol. Biol.* 166 557–580. 10.1016/S0022-2836(83)80284-8 6345791

[B19] Heilig H. G. H. J. Zoetendal E. G. Vaughan E. E. Marteau P. Akkermans A. D. L. De Vos W. D. L. (2002). Molecular diversity of *Lactobacillus* spp. and other lactic acid bacteria in the human intestine by specific amplification of 16S ribosomal DNA. *Appl. Environ. Microbiol.* 68 114–123. 10.1128/AEM.68.1.114-123.2002 11772617 PMC126540

[B20] Jangi S. Gandhi R. Cox L. M. Li N. von Glehn F. Yan R. (2016). Alterations of the human gut microbiome in multiple sclerosis. *Nat. Commun.* 7:12015 10.1038/ncomms12015 PMC4931233 27352007

[B21] Koutrolos M. Berer K. Kawakami N. Wekerle H. Krishnamoorthy G. (2014). Treg cells mediate recovery from EAE by controlling effector T cell proliferation and motility in the CNS. *Acta Neuropathol. Commun.* 2:163 10.1186/s40478-014-0163-1 PMC4268825 25476447

[B22] Kumar S. Stecher G. Tamura K. (2016). MEGA7: molecular Evolutionary Genetics Analysis version 7.0 for bigger datasets. *Mol. Biol. Evol.* 33 1870–1874. 10.1093/molbev/msw054 27004904 PMC8210823

[B23] Lee Y. K. Menezes J. S. Umesaki Y. Mazmanian S. K. (2011). Proinflammatory T-cell responses to gut microbiota promote experimental autoimmune encephalomyelitis. *Proc. Natl. Acad. Sci. U.S.A.* 108 4615–4622. 10.1073/pnas.1000082107 20660719 PMC3063590

[B24] Leonel A. J. Alvarez-Leite J. I. (2012). Butyrate: implications for intestinal function. *Curr. Opin. Clin. Nutr. Metab. Care* 15 474–479. 10.1097/MCO.0b013e32835665fa 22797568

[B25] Levast B. Li Z. Madrenas J. (2015). The role of IL-10 in microbiome-associated immune modulation and disease tolerance. *Cytokine* 75 291–301. 10.1016/j.cyto.2014.11.027 25542093

[B26] Li M. O. Flavell R. A. (2008). Contextual regulation of inflammation: a duet by transforming growth factor-beta and interleukin-10. *Immunity* 28 468–476. 10.1016/j.immuni.2008.03.003 18400189

[B27] Liu S. da Cunha A. P. Rezende R. M. Cialic R. Wei Z. Bry L. (2015). The host shapes the gut microbiota via fecal microRNA. *Cell Host Microbe* 19 32–43. 10.1016/j.chom.2015.12.005 PMC4847146 26764595

[B28] Lukic J. Strahinic I. Milenkovic M. Golic N. Kojic M. Topisirovic L. (2013). Interaction of *Lactobacillus fermentum* BGHI14 with rat colonic mucosa – implications for colitis induction. *Appl. Environ. Microbiol.* 79 5735–5744. 10.1128/AEM.01807-13 23851097 PMC3754154

[B29] Mielcarz D. W. Kasper L. H. (2015). The gut microbiome in multiple sclerosis. *Curr. Treat. Options Neurol.* 17 344 10.1007/s11940-015-0344-7 25843302

[B30] Miljkovic D. Stosic-Grujicic S. Markovic M. Momcilovic M. Ramic Z. Maksimovic-Ivanic D. (2006). Strain difference in susceptibility to experimental autoimmune encephalomyelitis between Albino Oxford and Dark Agouti rats correlates with disparity in production of IL-17, but not nitric oxide. *J. Neurosci. Res.* 84 379–388. 10.1002/jnr.20883 16676327

[B31] Miyake S. Kim S. Suda W. Oshima K. Nakamura M. Matsuoka T. (2015). Disbiosis in the gut microbiota of patients with multiple sclerosis, with a striking depletion of species belonging to Clostridia XIVa and IV clusters. *PLoS ONE* 10:e0137429 10.1371/journal.pone.0137429 PMC4569432 26367776

[B32] Momcilović M. Mostarica-Stojković M. Miljković D. (2012). CXCL12 in control of neuroinflammation. *Immunol. Res.* 52 53–63. 10.1007/s12026-012-8282-x 22392052

[B33] Ochoa-Repáraz J. Kasper L. H. (2016). The influence of gut-derived CD39 regulatory T cells in CNS demyelinating disease. *Transl. Res.* 10.1016/j.trsl.2016.07.016 [Epub ahead of print]. PMC5164971 27519147

[B34] Ochoa-Reparaz J. Mielcarz D. W. Ditrio L. E. Burroughs A. R. Foureau D. M. Haque-Begum S. (2009). Role of gut commensal microflora in the development of experimental autoimmune encephalomyelitis. *J. Immunol.* 183 6041–6050. 10.4049/jimmunol.0900747 19841183

[B35] Peris-Bondia F. Latorre A. Artacho A. Moya A. D’Auria G. (2011). The active human gut microbiota differs from the total microbiota. *PLoS ONE* 6:e22448 10.1371/journal.pone.0022448 PMC3145646 21829462

[B36] Presley L. L. Wei B. Braun J. Borneman J. (2010). Bacteria associated with immunoregulatory cells in mice. *Appl. Environ. Microbiol.* 76 936–941. 10.1128/AEM.01561-09 20008175 PMC2813032

[B37] Reeves A. E. Theriot C. M. Bergin I. L. Huffnagle G. B. Schloss P. D. Young V. B. (2011). The interplay between microbiome dynamics and pathogen dynamics in a murine model of *Clostridium difficile* infection. *Gut Microbes* 2 145–158. 10.4161/gmic.2.3.16333 21804357 PMC3225775

[B38] Rott O. Fleischer B. Cash E. (1994). Interleukin-10 prevents experimental allergic encephalomyelitis in rats. *Eur. J. Immunol.* 24 1434–1440. 10.1002/eji.1830240629 7515815

[B39] Sommer F. Bäckhed F. (2013). The gut microbiota - masters of host development and physiology. *Nat. Rev. Microbiol.* 11 227–238. 10.1038/nrmicro2974 23435359

[B40] Spor A. Koren O. Ley R. (2011). Unravelling the effects of the environment and host genotype on the gut microbiome. *Nat. Rev. Microbiol.* 9 279–290. 10.1038/nrmicro2540 21407244

[B41] Stanisavljević S. Lukic J. Momcilovic M. Miljkovic M. Jevtic B. Kojic M. (2016). Gut-associated lymphoid tissue, gut microbes and susceptibility to experimental autoimmune encephalomyelitis. *Benef. Microbes* 7 363–373. 10.3920/BM2015.0159 26839070

[B42] Stosic-Grujicic S. Ramic Z. Bumbasirevic V. Harhaji L. Mostarica-Stojkovic M. (2004). Induction of experimental autoimmune encephalomyelitis in Dark Agouti rats without adjuvant. *Clin. Exp. Immunol.* 136 49–55. 10.1111/j.1365-2249.2004.02418.x 15030513 PMC1808989

[B43] Suchodolski J. S. Markel M. E. Garcia-Mazcorro J. F. Unterer S. Heilmann R. M. Dowd S. E. (2012). The fecal microbiome in dogs with acute diarrhea and idiopathic inflammatory bowel disease. *PLoS ONE* 7:e51907 10.1371/journal.pone.0051907 PMC3530590 23300577

[B44] Telesford K. M. Yan W. Ochoa-Reparaz J. Pant A. Kircher C. Christy M. A. (2015). A commensal symbiotic factor derived from *Bacteroides fragilis* promotes human CD39(+)Foxp3(+) T cells and Treg function. *Gut Microbes* 6 234–242. 10.1080/19490976.2015.1056973 26230152 PMC4615798

[B45] Uzelac G. Miljkovic M. Lozo J. Radulovic Z. Tosic N. Kojic M. (2015). Expression of bacteriocin LsbB is dependent on a transcription terminator. *Microbiol. Res.* 179 45–53. 10.1016/j.micres.2015.06.011 26411894

[B46] Werner T. Wagner S. J. Martinez I. Walter J. Chang J. S. Clavel T. (2011). Depletion of luminal iron alters the gut microbiota and prevents Crohn’s disease-like ileitis. *Gut* 60 325–333. 10.1136/gut.2010.216929 21076126

[B47] Zhong Y. Nyman M. Fak F. (2015). Modulation of gut microbiota in rats fed high-fat diets by processing whole-grain barley to barley malt. *Mol. Nutr. Food Res.* 59 2066–2076. 10.1002/mnfr.201500187 26184884

